# No association between the wearing-off effect and α4-integrin receptor saturation in natalizumab treated patients with relapsing-remitting multiple sclerosis

**DOI:** 10.1016/j.neurot.2026.e00888

**Published:** 2026-03-20

**Authors:** Sean A. Freeman, Celso Rual, Damien Biotti, Marianne Lepetit, Juliette Le Berre, Kylynne Ferrara, Emmanuel Treiner, Jonathan Ciron

**Affiliations:** a Centre Ressources et Compétence Sclérose en Plaques, Département de Neurologie CHU PURPAN, Hôpital Pierre-Paul Riquet Place du Dr Baylac TSA 40031, 31059 TOULOUSE Cedex 9, France; b Toulouse Institute for Infectious and Inflammatory Diseases (Infinity), Inserm UMR1291, Université Toulouse III, Toulouse, France; c University of Toulouse, MetDatAS, CIC 1436, France; d Faculty of Medicine, University Toulouse III Paul Sabatier, Toulouse, France; e Laboratory of Immunology, University Hospital of Toulouse, Toulouse, France

**Keywords:** Multiple sclerosis, Natalizumab, Wearing-off effect, α4-integrin receptor saturation

## Abstract

Approximately half of patients with relapsing-remitting multiple sclerosis treated with natalizumab (NTZ) report transient symptom worsening - particularly fatigue - toward the end of the dosing interval. This phenomenon, commonly referred to as the wearing-off effect (WOE), has been hypothesized to result from a decrease in α4-integrin receptor saturation on circulating leucocytes. However, existing evidence is inconsistent, considering either small samples studied or conflicting results derived from previous studies. Our study aimed to investigate the association between α4-integrin receptor saturation and the WOE in a large prospective cohort of NTZ treated patients. We conducted a prospective observational study collecting demographic and clinical data, including WOE assessment via a structured questionnaire, treatment interval data, and α4-integrin receptor saturation levels across T-lymphocyte subset. Among 117 participants, 57.3 % reported experiencing WOE. WOE was significantly associated with higher disability (EDSS score) and longer NTZ treatment duration. However, no significant correlation was found between WOE and α4-integrin receptor saturation in any T-lymphocyte subset. Our findings suggest that WOE is not related to reduced α4-integrin receptor saturation and thus does not reflect diminished pharmacodynamic efficacy of NTZ. These results provide reassurance regarding the continued therapeutic effect of NTZ in patients experiencing WOE.

## Introduction

Natalizumab (NTZ) is a highly effective disease-modifying therapy for persons with relapsing-remitting multiple sclerosis (pwRRMS), reducing the frequency of clinical and radiological activity, and slowing disease progression [1]. Beyond its clear effect on inflammatory disease activity, natalizumab-treated patients sometimes report a sustained improvement in physical and psychological health following therapy initiation [2]. However, approximately 50 % of patients treated with NTZ report transient worsening of symptoms, particularly increased fatigue, at the end of their dosing cycle [3]. This “wearing-off effect” (WOE) is a clinically significant issue that may adversely affect patients’ quality of life.

An increasing number of neurologists are extending NTZ treatment intervals beyond the 4-week standard interval dosing (SID). Indeed, reassuring efficacy and safety data have been reported with an extended interval dosing (EID) regimen of 5 or 6 weeks [4,5]. Nevertheless, some studies have suggested that EID could increase WOE, arguing for individualized treatment regimens [6].

NTZ specifically binds the α4-integrin receptor on leukocytes, interfering with its ability to bind the VCAM-1 on vascular endothelial cells, thus preventing leukocyte migration through the blood-brain barrier. Αlpha4-integrin receptor saturation, defined as the level of NTZ bound to α4-integrin on leukocytes, is a potential biomarker to monitor and individualize NTZ therapy. There is conflicting evidence regarding the relationship between pharmacodynamic NTZ data and the occurrence of the WOE. It has been suggested that the WOE might be related to lower α4-integrin receptor saturation by NTZ [7], while another group found no such association [8].

Our study aimed to evaluate the frequency of WOE in a monocentric cohort of pwRRMS treated with NTZ, explore a potential association between the WOE and α4-integrin receptor saturation, and examine these data according to whether patients were treated in a SID or an EID regimen.

## Materials and methods

### Study design and population

We designed a monocentric, prospective and descriptive observational study, conducted in the MS expert center of the University Hospital of Toulouse, France. According to French law on ethics, patients were informed that their codified data will be used for the study and signed written consent. This study was approved by in accordance with the recommendations of the French National Commission for Informatics and Liberties (CNIL) and was declared compliant to the MR-004 (Méthodologie de référence 004) of the CNIL by the institutional board of University Hospital of Toulouse, reference RnIPH 2023-45.

Our study was conducted between October 2022 and April 2023. During this period, we recruited all patients over 18 years of age who had been diagnosed with RRMS and had received a minimum of either 6 intravenous infusions or subcutaneous injections of NTZ in the MS expert center of the University Hospital of Toulouse. Inclusion occurred on the day of the semestrial biological monitoring of the JC virus serology, and immediately prior to NTZ administration.

### Collection of clinical data

Assisted by the European Database for Multiple Sclerosis (EDMUS) software linked to the French Observatory of Multiple Sclerosis (OFSEP) database, we collected the following demographic and clinical characteristics from the patients’ medical records: age; sex; body mass index (BMI); disease duration (years since the diagnosis); NTZ treatment duration; subcutaneous or intravenous administration of NTZ; dosing interval; and last Expanded Disability Status Scale (EDSS) assessment.

Patients receiving NTZ at 5- or 6-week intervals were categorized as in the EID group, while patients on the standard 4-week interval were categorized as in the SID group.

Patients were questioned on the day of inclusion about their experience of WOE (supplemental data 1). They were informed that WOE is usually defined as “an increase of fatigue and/or an increase of the usual symptoms and/or a recurrence of symptoms in the last days before the subsequent administration of NTZ”. They were asked to choose a category among the following: never, sometimes, frequently or at each administration. The WOE group comprised patients having experienced WOE either “sometimes”, “frequently” or “at each administration”. Patients were also asked whether they experienced WOE at the time of the biological sampling on the day of their inclusion in the study.

### Collection of biological data

At inclusion, fresh blood was collected in EDTA-coated tubes immediately prior to NTZ administration and rapidly transferred to the laboratory of Immunology of the Toulouse University Hospital for analysis.

One hundred microliters of blood was transferred into three different tubes: blank (T1), test tube (T2) and saturation control (T3).

The saturation control was incubated with 20 μg/ml of NTZ for 15 min at room temperature (RT). After two washes in phosphate-buffered saline (PBS), the three tubes were incubated with a cocktail of fluorescent monoclonal antibodies (mAbs) (anti-CD45RO-PC7; Anti-CCR7-PE; Anti-CD3-AR700; Anti-CD8-PCP-Cy5.5; Anti-CD4-AF750; Anti-CD45-BV510), and the test and saturation controls additionally received an anti-IgG4-FITC mAb to reveal NTZ binding.

mAb Labeling was performed by incubation at RT for 15 min, followed by lysis of red blood cells using 2 ml FACS Lyse (BD biosciences), incubating for 15 min at RT, followed by washing in PBS before resuspending the cell pellets in 250 μl PBS. Data acquisition was carried out on a DxFlex automated system (Beckman Coulter) and data analysis was performed using Kaluza software (Beckman Coulter).

Saturation was calculated as follows:

$$\%saturation=\frac{\left(MFI\;FITC\;\left(T2\right)-MFI\;FITC\;\left(T1\right)\right)}{\left(MFI\;FITC\;\left(T3\right)-MFI\;FITC\;\left(T1\right)\right)}$$

MFI = median fluorescence.

The analysis was performed separately on the following T-lymphocyte populations: CD3^+^, CD4^+^, CD8^+^ T-lymphocytes, naïve CD4^+^ T-lymphocytes (CD3^+^CD4^+^CD45RA^+^CCR7^+^), CD4^+^ central memory (CD3^+^CD4^+^CD45RA^−^CCR7^+^), CD4^+^ effector memory (CD3^+^CD4^+^CD45RA^−^CCR7^-^), naive CD8^+^ T lymphocytes (CD3^+^CD8^+^CD45RA^+^CCR7^+^), CD8^+^ central memory (CD3^+^CD8^+^CD45RA^−^CCR7^+^), CD8^+^ effector memory (CD3^+^CD8^+^CD45RA^−^CCR7^-^), RA^+^ effector memory (EMRA) (CD3^+^CD8^+^CD45RA^+^CCR7^-^). CD4^+^ EMRA were excluded due their low numbers in the blood of most patients.

Preliminary experiments showed significant variability when samples were processed more than 16 h after drawing; thus, we only included patients when samples could be processed on the same day they were drawn. Repeatability was assessed and estimated to show a variation below 10 % in the saturation measurement. Some samples reached calculated values above 100 %, due to technical and analytical variations and were assigned to the value 100 %.

### Statistics

Descriptive analyses were performed to present the demographic and clinical characteristics of patients. Categorical variables were represented in percentages and frequencies, while continuous variables were presented as means and standard deviation. Chi-square test (χ2) and Fisher's exact test were employed to compare distributions of the WOE in relation to the categorical clinical variables. Wilcoxon rank-sum test and Kruskal-Wallis rank-sum test were used, where appropriate, for assessing quantitative variables. The statistical analyses were conducted and the results analyzed with regards to an alpha level of 0.05. All statistical analysis was performed using R (version 4.2.2).

#### Availability of data and materials

Anonymized patient data may be shared and made available by request from any investigator.

## Results

Of the 153 pwRRMS treated with NTZ in our center, 117 (76.4 %) were included in the study. The reasons for exclusion were sampling outside of the routine laboratory hours for analysis of α4-integrin receptor saturation (N = 31), and treatment with NTZ for less than 6 months (N = 5). Included patient characteristics and WOE determinants are presented in Table 1.

**Table 1:** Baseline demographics and clinical characteristics of patients according to reporting the wearing-off effect (WOE).

Variable	WOE – *N* = 50	WOE +*N* = 67	Overall *N* = 117	*p*-value^a^
**Age**				0.068
Mean (SD)	40.44 (9.55)	43.78 (10.23)	42.38 (10.04)	
**Sex, *N* (%)**				0.6
F	35 (70.00 %)	50 (74.63 %)	85 (72.65 %)	
M	15 (30.00 %)	17 (25.37 %)	32 (27.35 %)	
**BMI (kg/m^2^)**				0.14
Mean (SD)	24.21 (4.03)	23.39 (4.95)	23.72 (4.59)	
**Length of disease evolution (years)**				0.7
Mean (SD)	13.06 (7.71)	13.54 (7.70)	13.33 (7.67)	
**Number of treatment cycles**				**0.018**
Mean (SD)	59.60 (45.91)	79.87 (47.37)	71.21 (47.63)	
**Interval dosing in weeks, *N* (%)**				0.5
4	14.00 (28.00 %)	24.00 (35.82 %)	38.00 (32.48 %)	
5	15.00 (30.00 %)	21.00 (31.34 %)	36.00 (30.77 %)	
6	21.00 (42.00 %)	22.00 (32.84 %)	43.00 (36.75 %)	
**Interval dosing regimen, *N* (%)**				0.4
Extended	36 (72.00 %)	43 (64.18 %)	79 (67.52 %)	
Standard	14 (28.00 %)	24 (35.82 %)	38 (32.48 %)	
**EDSS**				**0.003**
Mean (SD)	1.75 (1.78)	3.17 (4.45)	2.56 (4.51)	
**Route of administration**				0.2
IV, n (%)	19 (38.00 %)	34 (50.75 %)	53 (45.30 %)	
SC, n (%)	31 (62.00 %)	33 (49.25 %)	64 (54.70 %)	

*p*-value of equal or less than 0.05 is considered significant.

a Wilcoxon rank sum test; Pearson's Chi-squared test. Bolded values indicate statistically significant values.

Sixty-seven (57.3 %) of the 117 included patients reported having previously experienced WOE, while 50 (42.7 %) reported never having experienced it. Among those who experienced WOE, 25 (37.3 %) had it at each administration, 25 (37.3 %) had it frequently, and 17 (25.4 %) experienced it only sometimes.

WOE appeared in a mean (SD) of 6.0 (2.3) days before the following NTZ cycle. Among patients receiving 4-week interval dosing, WOE appeared in a mean of 5.9 (1.5) days before the subsequent NTZ cycle, compared with 6.3 (3.0) days and 5.8 (2.5) days in patients with 5- and 6-week interval dosing, respectively. WOE disappeared in a mean of 2.0 (1.6) days after NTZ administration. There were no significant differences between SID and EID groups, in either the onset of the WOE prior to the next NTZ administration (5.9 days vs 6.2 days, respectively; *p =* 0.6) (Supplementary Fig. 1), or the time to WOE resolution following NTZ administration (1.9 days vs 2.0 days, respectively; *p* > 0.9).

**Fig.1:**
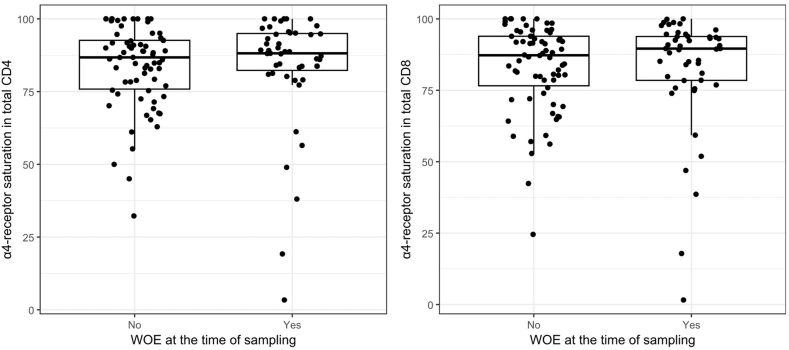
Boxplot comparison of the α4-integrin receptor saturation (by lymphocyte subset) with the presence or absence of the WOE at the time of sampling.

At the time of sampling, among the 117 patients, 70 did not experience a WOE, whereas 47 did. No relation was found between α4-integrin receptor saturation in any T-lymphocyte subset and the occurrence of WOE at the time of sampling (Fig. 1, showing results in total CD4^+^ subset and in total CD8^+^ subset; Table 2, showing results in all T-lymphocyte subsets). Mean CD4^+^ lymphocyte α4-integrin receptor saturation was 84.4 % ± 19.0 % in patients with WOE and 84.5 % ± 13.8 % in patients without WOE (*p =* 0.4). Mean CD8^+^ lymphocyte α4-integrin receptor saturation was 83.5 % ± 19.7 % in patients with WOE and 83.8 % ± 14.8 % in patients without WOE (*p =* 0.6).

**Table 2:** Comparison of α4-integrin receptor saturation (by lymphocyte subset) with the presence or absence of the WOE at the time of sampling.

Variable	No WOE, *N* = 70	WOE *N* = 47	*p*-value
**CD3^−^**			0.7
Mean (SD)	83.16 (14.31)	82.26 (19.36)	
**CD3^+^**			0.3
Mean (SD)	83.62 (14.10)	84.04 (19.41)	
**Total CD4^+^**			0.4
Mean (SD)	84,49 (13.81)	84,37 (18.97)	
**CD4^+^CM**			0.7
Mean (SD)	84.11 (13.83)	83.51 (18.87)	
**CD4^+^EM**			0.7
Mean (SD)	84.50 (14.10)	83.20 (20.30)	
**CD4^+^N**			0.7
Mean (SD)	84.50 (13.72)	83.76 (18.17)	
**Total CD8^+^**			0.6
Mean (SD)	83.84 (14.77)	83.46 (19.66)	
**CD8^+^CM**			0.6
Mean (SD)	82.03 (14.67)	81.57 (19.50)	
**CD8^+^EM**			0.7
Mean (SD)	82.85 (15.14)	82.18 (19.37)	
**CD8^+^EMRA**			0.089
Mean (SD)	84.28 (14.88)	85.88 (20.16)	
**CD8^+^N**			0.076
Mean (SD)	82.29 (15.29)	84.25 (20.49)	

Patients who experienced WOE symptoms had significantly more treatment cycles than patients who never experienced such symptoms (79.87 versus 59.60, respectively; *p =* 0.018) (Table 1). They also had a significantly greater disability, as measured by a higher EDSS score (3.17 versus 1.75, respectively; *p =* 0.003). No significant differences were found between the two groups regarding sex, age, BMI and interval dosing. The route of administration of NTZ (intravenous versus subcutaneous) was not associated with the occurrence of the WOE (*p =* 0.2).

**Fig.2:**
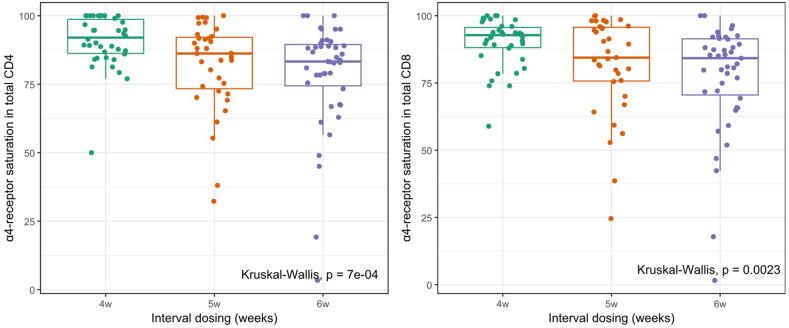
Boxplot comparison of the α4-integrin receptor saturation (in total CD4^+^ and total CD8^+^ subsets) by interval dosing.

**Fig.3:**
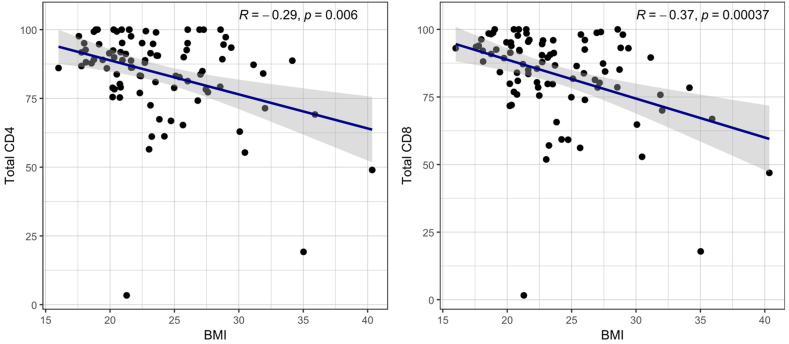
Scatter plot, correlation of BMI with α4-integrin receptor saturation in total CD4^+^ and total CD8^+^ subsets.

As expected, α4-integrin receptor saturation was significantly lower in the EID group than in the SID group, in all lymphocyte subsets analyzed (Table 3). In the EID group, α4-integrin receptor saturation was lower with an interval dosing at 6 weeks than with an interval dosing at 5 weeks (Fig. 2). BMI was inversely correlated with α4-integrin receptor saturation (CD8^+^ *R* = − 0.37; *p =* 6.3*e*^−5^ while in total CD4^+^ *R* = − 0.33; *p =* 0.00046) (Fig. 3).

**Table 3:** Mean α4-integrin receptor saturation (by lymphocyte subset) in standard interval dosing and extended interval dosing. Bolded values indicate statistically significant values.

Variable	Extended, *N* = 79	Standard, *N* = 38	*p*-value
**CD3^−^**			**0.002**
Mean (SD)	79.56 (18.20)	89.54 (9.02)	
**CD3^+^**			**<0.001**
Mean (SD)	80.58 (17.93)	90.47 (9.70)	
**Total CD4^+^**			**<0.001**
Mean (SD)	81.29 (17,51)	90.99 (9.56)	
**CD4^+^CM**			**0.001**
Mean (SD)	80.55 (17.83)	90.77 (7.54)	
**CD4^+^EM**			**<0.001**
Mean (SD)	80.74 (18.27)	90.85 (10.49)	
**CD4^+^N**			**0.005**
Mean (SD)	81.56 (17.34)	89.71 (9.05)	
**Total CD8^+^**			**0.005**
Mean (SD)	80.60 (18.76)	90.11 (9.06)	
**CD8^+^CM**			**0.013**
Mean (SD)	79.19 (18.35)	87.37 (10.86)	
**CD8^+^EM**			**0.004**
Mean (SD)	79.42 (18.82)	89.16 (9.06)	
**CD8^+^EMRA**			**0.003**
Mean (SD)	82.03 (18.83)	90.93 (10.87)	
**CD8^+^N**			**0.014**
Mean (SD)	80.31 (19.30)	88.83 (11.21)	

*p*-value of equal or less than 0.05 is considered significant.

## Discussion

Here, we report to our knowledge the largest prospective study population demonstrating that WOE under NTZ therapy for pwRRMS is not associated with the degree of α4-integrin receptor saturation, regardless of the lymphocyte subset.

Literature exploring the relationship between WOE and the pharmacokinetics/pharmacodynamics of NTZ is scarce. In our cohort, WOE is frequently reported, with 57.3 % of all patients having experienced it, consistent with data from other studies [3]. Our findings align with the study by van Kempen et al., wherein over half of the patients had experienced WOE, yet neither α4-integrin receptor saturation nor NTZ concentration was associated with the occurrence of this phenomenon [8]. In contrast, in a small study of 40 patients treated with NTZ, Bringeland et al. observed lower α4-integrin receptor saturation in a very limited number of patients who regularly experienced WOE (n = 8) [7]. However, they found no relation between α4-integrin receptor saturation and the occurrence of WOE in the group of patients who experienced only sometimes WOE [7].

We used the same methodology than Van Kempen et al., which differs from Bringeland et al., suggesting that technical issues could be involved in the observed discrepancies between studies. However, although our technique may over-estimate receptor occupancy levels [9], this methodological aspect involves all of our patients and does not explain the absence of correlation between the WOE and the α4-integrin receptor saturation levels in our study.

While no biological determinant of WOE has been identified, three main hypotheses regarding the mechanisms of WOE can be proposed. The first hypothesis is that when α4-integrin receptor saturation is below a threshold (which remain to be determined), some lymphocytes could cross the blood-brain barrier (BBB), leading to cytokine production and low-grade inflammation into the central nervous system. According to this hypothesis, a lower α4-integrin receptor saturation is expected to be observed in the WOE group; however, our findings do not support this hypothesis. Additionally, patients with WOE usually report symptom improvement almost immediately after receiving NTZ (within a median of 2 days in our study), thus a timeframe too short to be related to a direct biological effect of NTZ, as lymphocytes that had previously crossed the BBB are not immediately removed [10].

Another possible mechanism of WOE is that NTZ may affect the peripheral release of soluble factors such as cytokines. The cytokine balance, specifically the Th1/Th2 ratio, potentially plays a role in the pathophysiology of fatigue in patients with MS [11]. However, the literature on this topic is limited, and some studies have failed to show a peripheral anti-inflammatory action of NTZ [12,13]. Moreover, soluble cytokines are influenced by many factors, making it challenging to prove this hypothesis.

Lastly, a third hypothesis regarding the WOE is that it may, at least partially or largely reflect a placebo/nocebo effect, in which the perceived improvement in symptoms after infusion or injection might be attributable to the expectation of symptom improvement.

Interestingly, there was no difference in the frequency of WOE between the SID and EID groups in our cohort. Nevertheless, this result contrasts with a retrospective study by Bernardes and colleagues, which found that the prevalence and duration of WOE increased with EID [6]. Addressing this question would require randomization and blinding of dosing intervals to determine whether EID is really associated with WOE symptoms.

In our cohort, when evaluating the disease features, WOE was only associated with a longer treatment duration and higher EDSS score at baseline, suggesting a relation with disability status in term of disease evolution rather than a relation with disease activity.

As expected, we found that α4-integrin receptor saturation by NTZ was lower in the EID group than in the SID, most likely due to progressive desaturation of the receptor over time. Our study also showed an inverse correlation between α4-integrin receptor saturation and BMI, consistent with findings from previous studies [14,15]. Not all patients with low saturation in our study had high BMI; a previous study reported that body weight only partially predicts variability in α4-integrin receptor saturation, suggesting that other factors, such as density and turnover of α4β1-integrin, may drive this variability [16].

Our study has some limitations. Almost 25 % of the patients treated with NTZ in our center were excluded from the study, most of them because of sampling outside of the routine laboratory hours for the specific analysis of α4-integrin receptor saturation, in the real-life setting of our cohort. Thus, we cannot rule out a sampling bias. Moreover, we chose to divide patients into 2 groups, according to the presence or absence of WOE. However, the WOE group was quite heterogenous, ranging from patients who experienced the WOE only sometimes, to patients who experienced it at each administration. Additionally, we deliberately focused our study on T cells, in accordance with the methodological framework described by Van Kempen et al. [8], and supported by the substantial body of evidence highlighting the central role of T cells in the pathophysiology of MS. Consequently, our conclusions specifically pertain to T-cell biology. Other leukocyte subsets, including B cells, may theoretically mediate WOE, and further investigation into the relationship between WOE and additional leukocyte populations is warranted. Lastly, we cannot rule out a confirmation bias, given the methodology of our study, relying on directed questions about a range of symptoms.

Our study underlines that WOE is not related to α4-integrin receptor desaturation. This important result provides reassuring data showing that patients experiencing the WOE are not undertreated.

## Author contributions

**Sean A. Freeman**: First Author, Principal Investigator; data management. **D. Biotti**: Corresponding Author, neurological management, data management; **Celso Rual**: neurological management, data management; **Marianne Lepetit**: statistics; **Kylynne Ferrara**: neurological management; **Emmanuel Treiner**: immunological testing, data management**, Jonathan Ciron**: neurological management, data management.

## Funding

The authors received no financial support for the research, authorship and/or publication of this article.

## Declaration of competing interests

The authors declare that they have no known competing financial interests or personal relationships that could have appeared to influence the work reported in this paper.

## Figures and Tables

**Figure:**
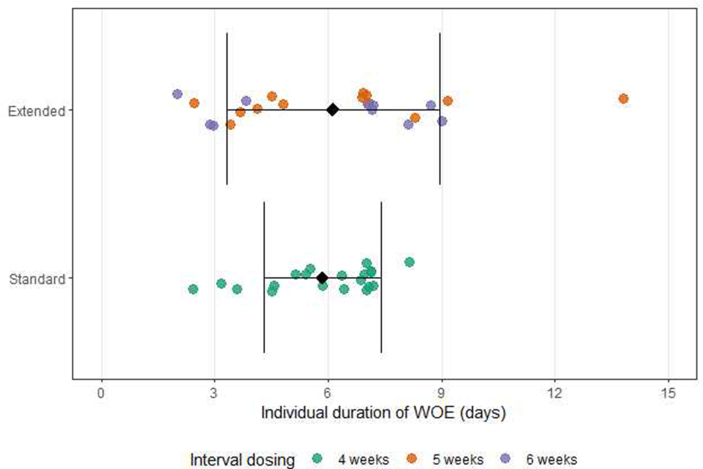
**Supplementary figure 1**: Strip plot showing individual delays to wearing-off effect (WOE) prior to the subsequent natalizumab administration according to dosing regimen. Each dot represents one patient. Colors indicate inter-dose intervals (4, 5, and 6 weeks).
