# Supplementary material for: No association between the wearing-off effect and α4-integrin receptor saturation in natalizumab treated patients with relapsing-remitting multiple sclerosis
Source: Neurotherapeutics. 2026 Mar 20;23(2):e00888. doi: 10.1016/j.neurot.2026.e00888 (PMC13069423; doi:10.1016/j.neurot.2026.e00888)
Supplement: Multimedia component 1 — . [file mmc1.docx]

**Questionnaire on the Wearing-off Effect of Natalizumab**

The neurology and immunology departments of the Multiple Sclerosis Resource and Expertise Center (CRC) at Toulouse University Hospital (CHU de Toulouse) wish to study the prevalence of the wearing-off effect experienced by some patients treated with Natalizumab as the next treatment administration approaches.

This effect corresponds to a sensation of worsening at a distance from the last treatment administration, while the time for the next administration has not yet arrived, even though it is approaching.
This effect therefore causes the patient to feel a need not to delay renewing the treatment in order to regain a better neurological state.

The team also wishes to investigate the existence of a correlation between this wearing-off effect and the level of saturation of Natalizumab at its target (Natalizumab binds to the alpha4 subunit of the alpha4beta1 receptor protein located on the surface of lymphocytes).

The blood test performed every 6 months to monitor JC virus serology will therefore now also include measurement of the saturation level of the alpha4beta1 receptor protein by Natalizumab.

The following questionnaire is proposed to investigate the occurrence of the wearing-off effect:

**1)** Do you generally experience a wearing-off effect (increased fatigue, worsening of the usual symptoms of the disease, reappearance of symptoms otherwise absent) in the last days before Natalizumab is re-administered?

a) Yes, every time ☐
b) Yes, frequently ☐
c) Sometimes ☐
d) No ☐

**2)** If yes to question 1 (answer a, b or c), do you experience it today?
…………………………….

**3)** If yes to question 1 (answer a, b or c), how many days before treatment re-administration does this effect generally begin? (Free response in days)
…………………………….

**4)** If yes to question 1 (answer a, b or c), how long after treatment administration does the effect disappear? (Free response in days)
…………………………….

**Last name:** ……………………………..
**First name:** ………………………….
